# Characterization and Genome Analysis of *Fusarium oxysporum* Provides Insights into the Pathogenic Mechanisms of the Pokkah Boeng Disease in China

**DOI:** 10.3390/microorganisms13030573

**Published:** 2025-03-03

**Authors:** Wenfeng Lin, Chi Zhang, Sehrish Akbar, Suyan Wu, Yabing Yue, Gege Wang, Yu Zhou, Charles A. Powell, Wei Yao, Jianlong Xu, Baoshan Chen, Muqing Zhang, Yixue Bao

**Affiliations:** 1 State Key Lab for Conservation and Utilization of Subtropical Agric-Biological Resources & Guangxi Key Lab for Sugarcane Biology, Guangxi University, Nanning 530004, China; 1224540633@163.com (W.L.); 18740375350@163.com (C.Z.); sehrishakbar746@gmail.com (S.A.); 18208262822@163.com (S.W.); 17740258753@163.com (Y.Y.); wanggegeiuaoa@163.com (G.W.); 18378692818@163.com (Y.Z.); yaoweimail@163.com (W.Y.); chenyaoj@gxu.edu.cn (B.C.); 2 Indian River Research and Education Center-Institute of Food and Agricultura, University of Florida, Fort Pierce, FL 34945, USA; capowell@ufl.edu; 3 Institute of Crop Sciences, Chinese Academy of Agricultural Sciences, Beijing 100081, China; xujianlong@caas.cn; 4 Hainan Yazhou Bay Seed Laboratory, Sanya 572024, China

**Keywords:** *Fusarium oxysporum*, Pokkah Boeng disease, pathogenic mechanism, disease prevention and control

## Abstract

Pokkah Boeng Disease (PBD) is a severe and devastating disease that causes significant damage and yield losses in China. The pathogenic fungus *Fusarium oxysporum* is responsible for the rapid onset of top rot symptoms in sugarcane. In this study, we selected a representative strain, BS2-6, to perform morphological observations of colonies and determine pathogenicity. We examined the effects of BS2-6 infestation on the ultrastructure of sugarcane leaves. Moreover, we sequenced the whole genome of BS2-6 and examined the effects of various nitrogen sources and chemical reagents on its growth and pathogenicity. Our results indicate that sugarcane leaves inoculated with BS2-6 quickly succumb to heart leaf and growing rot. Ultrastructural analysis revealed that the surface tissues of the diseased leaves were destroyed with mycelium, and conidia blocked leaf stomata, which ultimately led to the degradation of leaf tissues. Ammoniacal nitrogen significantly promoted mycelial growth, pigment secretion, and the expression of genes related to secondary metabolite synthesis, thereby accelerating the development of PBD. In addition, we found that carbendazim effectively inhibited the growth of BS2-6 at various concentrations. These findings provide important insights for the effective prevention and control of PBD during sugarcane production.

## 1. Introduction

Sugarcane is one of the world’s largest sugar crops, accounting for over 90% of sugar production and cultivation in China. Pokkah Boeng Disease (PBD) is a century-old disease that poses one of the most serious threats to sugarcane, causing significant damage and yield losses. The disease predominantly affects the tender leaves, leaf sheaths, and stalks at the apex of the sugarcane plant, resulting in leaf distortion, yellowing, the emergence of reddish-brown stripes, and perforations. In severe instances, it can cause necrosis and the decay of the young leaves at the sugarcane tip, ultimately leading to the wilting and death of the entire plant. The onset of this disease is associated with multiple factors, including monoculture planting, weak disease resistance, high temperature and humidity conditions, excessive nitrogen fertilization, and inadequate field management practices. In recent years, PBD has spread rapidly in China in all seasons, including winter. Guangxi, the largest sugarcane planting and production area in China, was initially reported to be severely impacted by PBD [1]. This disease is caused by the various species of *Fusarium* [2,3].

*Fusarium oxysporum* (*F. oxysporum*) has been reported to cause destructive vascular wilt and root rot in over 120 plant species [4,5]. Interactions between plants and pathogens have been reported to have various negative effects on plant growth and development [6]. The fungus penetrates the host roots directly through specialized hyphae and colonizes the cortex via both intracellular and intercellular growth. Previously, we conducted a disease survey and reported that *F. oxysporum* causes sugarcane PBD [3]. However, the impact of *F. oxysporum* infestation on the organization of sugarcane leaves as well as on the overall growth and development of sugarcane has not been reported.

Due to the advancement of sequencing technology and bioinformatics approaches, exploring the fungal development, systematics, and evolution at the molecular level has become more feasible [7]. However, the complete genome sequences for some *Fusarium* strains are still not available. Additionally, previous studies on *Fusarium* have primarily focused on phylogenetic lineages and functional gene clusters [8]. Therefore, there is a need to characterize the genomes of more *Fusarium* species and analyze their pathogenicity mechanism. *Fusarium oxysporum* f. sp. *lycopersici* strain 4287 (FOL4287), causing tomato wilt disease, was the first characterized genome of an isolate *F. oxysporum* strain. Its whole genome sequence has been widely used for comparative genomic analysis with other genomes [9]. However, there is currently no publicly available genome-wide information that exists on *F. oxysporum*, the causal agent of sugarcane PBD, which significantly limits the research on this disease. In addition, China is the world’s largest producer and consumer of chemical fertilizers. It accounts for nearly 30% of global fertilizer consumption on less than 9% of the world’s arable land. Inappropriate fertilizer usage, including over-application and a poor matching of fertilizers with crop needs, has led to a low utilization rate [10]. Under high temperature and humidity conditions, excessive nitrogen fertilizer use can cause sugarcane to grow too vigorously, leading to tender tissue that is more susceptible to infection by *Fusarium* sp. [11]. This significantly increases the incidence of sugarcane PBD. To effectively prevent and control this disease, it is necessary to take effective measures, such as rational fertilization and chemical control, to minimize the occurrence of PBD.

The present study is conducted to analyze the effects of *F. oxysporum* on the ultrastructure of sugarcane leaves. It also acquires extensive molecular data for a genome-wide characterization of *F. oxysporum* as well as the elucidation of methods for controlling the disease in the field through the rational application of nitrogen fertilizer and chemical agents. These findings will provide a basis for further research into the pathogenic mechanism of *F. oxysporum* and develop effective strategies for disease prevention and the breeding of disease-resistant sugarcane.

## 2. Materials and Methods

### 2.1. Strain Culture and Morphological Observation

The wild-type strain *F. oxysporum* BS2-6 was collected from Guangxi, Baise (by our group in 2015); coordinates: E 106°62′, N 23°90′. This strain was cultured on potato dextrose agar (PDA) medium (Hopebio, Qingdao, China) at 28 °C (2 to 3 days) for phenotypic observation and conidial collection. Strain BS2-6 was inoculated on both PDA and Carnation Leaf Agar (CLA) media (prepare a custom medium with fresh carnation leaves and agar), incubated at 28 °C for 5 to 7 days under 12 h light/dark, and the humidity was between 85% to 90% (three replicates per treatment). The morphological characteristics of the colonies were then observed. An appropriate amount of mycelium was collected from the medium to prepare a slide. The strain’s morphology, including conidia and chlamydospores, was observed using a 40× microscope.

### 2.2. Pathogenicity Assay

One-month-old sugarcane (variety zhongzhe1) was selected for the pathogenicity assay. A total of 100 μL (10^7^ CFU/mL) of BS2-6 conidial suspension (containing 0.05% Tween 20) (Thermo Fisher Scientific, Shanghai, China) was sprayed onto the leaf centers of the seedlings using a high-pressure spray gun (five replicates per treatment, on different individual plants). Sterile water was used as the control treatment. The onset of disease in the sugarcane was observed and recorded after 3 d. From the diseased leaves, pathogens were isolated and compared with the original pathogen in terms of colony morphology, mycelium color, and conidium morphology, completing the verification of Koch’s postulates.

### 2.3. Ultrastructure Analysis Using Scanning Electron Microscopy (SEM)

In the pathogenicity assay, the symptomatic leaf tissues were dissected 3 d after inoculation. For the paraffin section, samples were fixed with the FAA fixative (Servicebio, Wuhan, China) at room temperature. For scanning electron microscopy (SEM), samples were fixed with an electron microscopy fixative (Servicebio G1102, Wuhan, China) for 2 h at room temperature, then stored at 4 °C for later use. The slices were sectioned using a Leica UC7 ultrathin slicer, with a slice thickness in the range of 60–80 nm, and then the slices were transferred onto a 150-mesh square waffle film copper mesh. For microscopic observation, an orthogonal microscope (NIKON Eclipse ci; NIKON, Tokyo, Japan) was utilized to capture and analyze images for electron microscopy. For electron microscopy, the sample section was placed on a small copper mesh and positioned under a scanning electron microscope (HITACHI SU8100; Hitachi, Tokyo, Japan) for observation. A typical area was selected to be photographed. The field of view was first located at 40× magnification, followed by an observation at 100× and then at 400× magnification to detect subtle differences in the sections. A total of 15–20 fields of view were counted for each sample, and images were captured and saved at each magnification [12].

### 2.4. Reponses to Chemical Compounds In Vitro

Seven chemical compounds (mancozeb, copper 8-hydroxyquinoline, carbendazim, thiophanate-methyl, triadimefon, validamycins, and myclobutanil) were purchased from Sigma-Aldrich Co., Ltd. (Sigma-Aldrich, MO, USA). Different concentrations (10 ppm, 50 ppm, and 100 ppm) of the chemical compounds were prepared by dissolving the requisite quantity of each compound in PDA medium, and then we inoculated BS2-6 isolate. Each treatment was performed in triplicate, with a non-supplemented PDA medium as the control. The plates were incubated at 28 °C for 5 days in darkness and the diameter of the fungal colony was measured. The inhibitory rate was calculated as follows: % inhibition = [(Gc − Gt)/Gc] ×100, where Gc = Growth in control and Gt = Growth in treatment.

### 2.5. Genome Sequencing, Assembly, and Evaluation

The gDNA from the wild-type strain BS2-6 was extracted using the CTAB method [13]. Whole genome sequencing of BS2-6 was performed using the PacBio sequencing platform and Illumina sequencing platform. The filtered subreads were corrected using Canu v1.5 [14], followed by assembly using wtdbg v2.0 [15]. The assembled genome was then further corrected using Pilon v1.22 [16], resulting in a genome of higher accuracy. The completeness and accuracy of the assembled genomes were evaluated based on the Benchmarking Universal Single-Copy Orthologs (BUSCO) v5.1.2 (eukaryota_odb9) and RNA-seq data [17].

### 2.6. Gene Prediction

The repeat sequence database of the BS2-6 genome was constructed using LTR_FINDER v1.05 [18], MITE-Hunter v1.0 [19], RepeatScout v1.0.5 [20], and PILER-DF v2.4 [21]. This database was classified with PASTEClassifier v2.0 [22] and subsequently merged with the Repbase database to create the final repeat sequence database [23]. Finally, RepeatMasker v4.0.6 was used to predict the repetitive sequences of BS2-6 based on the constructed repeat sequence database [24].

An *ab* initio prediction was constructed using Augustus v2.4 [25], GlimmerHMM v3.0.4 [26], and SNAP v8.0.0 [27]. Additionally, a homologous species-based prediction was performed using GeMoMa v1.3.1 [28], while a transcriptome data-based prediction utilized PASA v2.4.1 [29]. The final integration of the results from these three prediction methods was performed using EVM v1.1.1 [30]. tRNAs in the genome were predicted using the software tRNAscan-SE v2.0 [31]. However, rRNAs in the genome as well as other ncRNAs besides tRNAs and rRNAs were predicted using the software Infernal v1.1 [32] based on the Rfam [33] database.

### 2.7. Gene Annotation

The predicted gene sequences were compared to functional databases using BLAST v2.2.31 [34], including KOG [35], KEGG [36], Swiss-Prot, TrEMBL [37], and Nr [38]. Based on the Nr database results, the application software Blast2GO v5.2.5 was used to perform a functional annotation of the GO database [39,40]. The software hmmer v3.3.2 was utilized to perform a Pfam functional annotation based on the Pfam database [41,42]. The protein sequences of the predicted genes were used to perform a BLAST comparison with the TCDB [43] and PHI [44] database to obtain the corresponding annotation results. The functional annotation of carbohydrate enzyme genes was performed using hmmer v3.3.2 software based on CAZyme [45]. Furthermore, the protein sequences of all predicted genes were analyzed using the software SignalP v4.0 to identify proteins containing signal peptides [46]. The protein sequences of all predicted genes were analyzed using the software TMHMM v2.0 to identify transmembrane proteins [47]. Proteins containing transmembrane helices were removed from the above-predicted signal peptide-containing proteins, and the remaining proteins were the secreted proteins. Secreted proteins were further analyzed using EffectorP v3.0 to predict effector proteins [48]. Lastly, we employed the iTAK1.0 program to identify transcription factors (TFs) among the gene models [49].

### 2.8. Phylogenetic Tree Construction

A phylogenetic tree was built by the protein sequences of selected *Fusarium* species (*F. oxysporum* BS2-6, *F. oxysporum* FOL4287, *F. oxysporum* Fo47, *F. proliferatum* ET1, *F. verticillioides* 7600, *F. fujikuroi* IMI58289, *F. mangiferae* MRC7560, *F. graminearum* PH-1, *F. pseudograminearum* CS3096, and *F. solani* SB1), including *Aspergillus nidulans* FGSC A4 and *Neurospora crassa* OR74A as an outgroup. The whole-protein sequences were aligned by MUSCLE v3.8.31 (http://www.drive5.com/muscle/, accessed on 14 January 2023) with default parameters. Phylogeny was performed using the maximum likelihood (ML) algorithm with the JTT amino acid substitution model implemented by phyML v4.0 [50].

### 2.9. Comparative Genome Analysis

The sequences of *F. oxysporum* BS2-6 and *F. oxysporum* FOL4287 were BLAST v2.2.31 aligned to obtain collinearity at the nucleic acid level according to the position of the homologous genes on the genome. TBtools v2.09 software was used to draw a collinearity map at the chromosome level of the two strains [51]. Protein sequences of four genomes (*F. oxysporum* BS2-6, *F. fujikuroi* IMI 58289, *F. verticillioides* 7600, and *F. oxysporum* FOL4287) were classified with OrthoMCL v2.0 software to identify the unique secondary metabolic families and functional gene clusters shared by the strains [52]. Adobe illustrator v1.0 (AI) software was used to draw microcollinear vector diagrams of functional gene clusters based on their gene sequence and position in the genome [53].

### 2.10. Transcriptome Analysis

BS2-6 spores were inoculated into 200 mL Erlenmeyer flasks containing 60 mL of potato dextrose water (PDW) medium (Hopebio, Qingdao, China), Czapek medium (per liter: K_2_HPO_4_ 1 g, MgSO_4_·7H_2_O: 0.5 g, KCl: 0.5 g, Fe_2_SO_4_: 0.01 g, and sucrose: 30 g) supplemented with 3 g/L of NaNO_3_, (NH_4_)_2_SO_4_ or urea as the nitrogen source, respectively (Hopebio, Qingdao, China). The flasks were shaken on a rotary shaker at 200 rpm at 28 °C for five days. Mycelia from the different nitrogen cultures were harvested by centrifugation at 8000 rpm for 10 min at 4 °C for the immediate extraction of total RNA. Total RNA was isolated from the harvested mycelia samples using the TRIzol^®^ Reagent (Invitrogen, Carlsbad, CA, USA) according to the manual instructions. RNA quality was verified using SmartSpec Plus (Thermo, Waltham, MA, USA). The library products were sequenced using an Illumina Nove-seq 6000 platform from the Biomarker company (Biomarker, Beijing, China). In total, RNA-seq libraries generated about 117 million reads. To obtain a clean read, the data were filtered with SeqPrep (https://github.com/jstjohn/SeqPrep, accessed on 14 November 2022) and Sickle (https://github.com/najoshi/sickle, accessed on 14 November 2022) software. The remaining clean reads were aligned to the reference genome (BS2-6) by HISAT2 v2.1.0 [54]. For quantification, the level of gene expression was calculated based on the fragments per kilobase of exon per million fragments mapped (FPKMs) using Cufflinks v1.0 (a threshold for the false discovery rate: FDR < 0.01; an absolute value of log_2_Ratio ≧ 2) [55]. Functional annotation by gene ontology terms was analyzed by Blast2GO v5.2.5 program GO [39,40]. KEGG pathway annotations were performed using BlastAll v1.0 software against the KEGG pathway database [36].

## 3. Results

### 3.1. Colony Morphology and Determination of Pathogenicity

**Figure 1:**
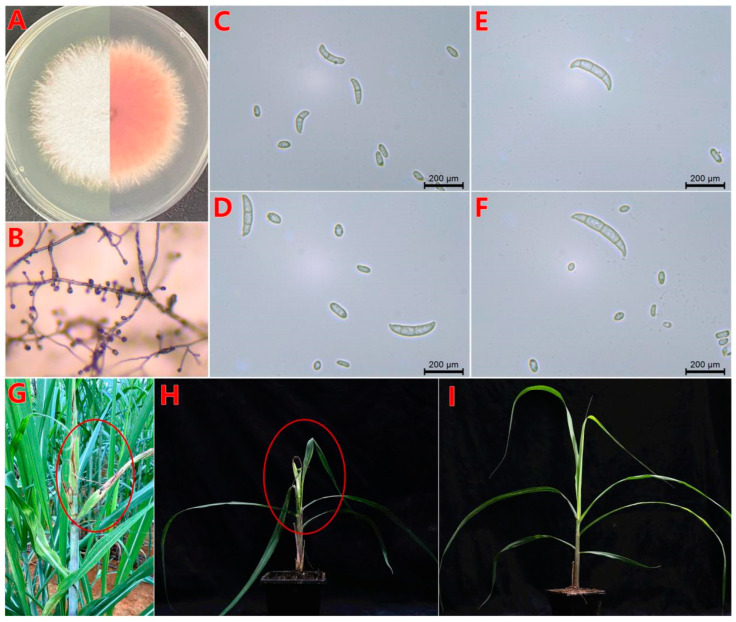
Symptoms of PBD on sugarcane and morphological characteristics of strain BS2-6. (**A**) Mycelium colony on PDA medium at 28 °C after 7 days of incubation (top view and bottom view); (**B**) microconidia in situ on carnation leaf-piece agar medium; (**C**–**F**) oval-to-kidney-shaped microconidia and macroconidia are slightly sickle-shaped, with a tapered apical cell and foot-shaped basal cell; (**G**) typical top rot of leaves in field sugarcane, the PBD phenotype in the field is marked by the red circle; (**H**) pathogenicity test on sugarcane leaf with BS2-6, the phenotypic manifestation of the PBD occurs within the red circle after inoculation and control water (**I**). Note: compare the phenotypes of sugarcane leaves after 7 d of infection.

The *F. oxysporum* BS2-6 strain produced abundant aerial mycelium on the PDA medium, exhibiting irregular margins with a distinct pink pigmentation that intensified with time (Figure 1A). Observations of the CLA medium showed that the microconidia consisted of multiple pseudopodia (Figure 1B). Under microscopy (40×), the macroconidia had 3–5 septa, was thin walled, and sickle shaped. However, the microconidia were rod-shaped or ovoid, with flattened bases, featuring 0–1 septa (Figure 1C–F).

After three days of infection with BS2-6, the leaves appeared wrinkled and twisted, with obvious reddish-brown striped spots. Subsequently, the heart-shaped leaves and growing points rotted and eventually died. The young tissues at the tip formed a top rot shape, which was consistent with the onset of symptoms in the field. There was no obvious difference between the control and healthy sugarcane leaves (Figure 1G–I). The PBD symptoms of sugarcane and the standardized criteria for disease assessment are based on the previous research [56]. The isolates obtained from the diseased leaves were similar with the colony, micro-, and macro- conidial morphologies of the inoculated strain BS2-6.

### 3.2. Effect of F. oxysporum BS2-6 on the Ultrastructure of Sugarcane Leaves

**Figure 2:**
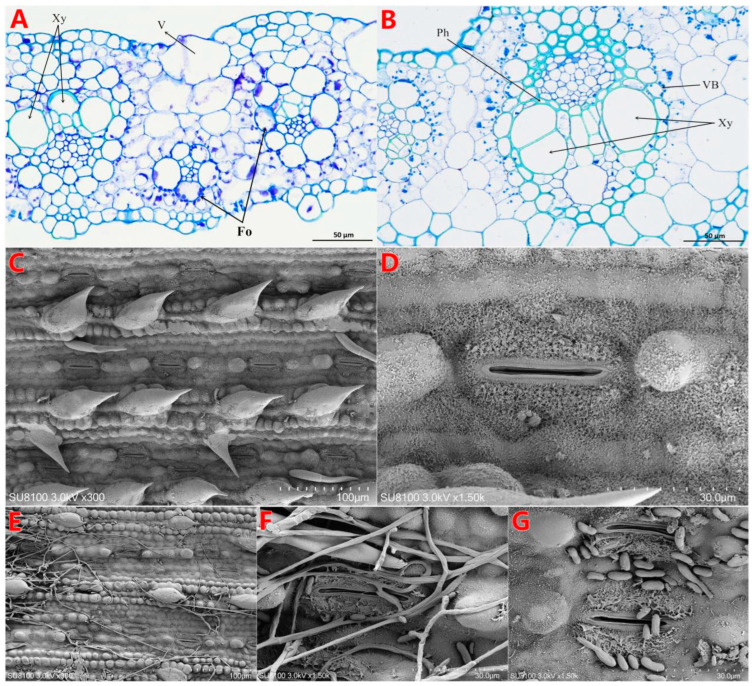
Cell structure of healthy and pathological changes in PBD sugarcane. (**A**) The structure of sick leaves is a bubble cell bulge; xylem growth is inhibited. (**B**) The tissue vascular bundle structure of healthy leaves is normal. (**C**,**D**) Scanning electron microscopy shows that healthy leaves have a regular tissue structure, a clearly visible shape, and normal stomata. (**E**–**G**) The tissue of infested leaves is destroyed, and mycelia and conidia have blocked the stomata of the leaves. Note: V—vacuole; Xy—xylem; Fo—*Fusarium oxysporum*; Ph—phloem; VB—vascular bundle.

The anatomical microstructure of sugarcane leaves under infection with BS2-6 revealed changes in the cell wall structure, the presence of pathogenic fungi within degraded leaf cells, bulging of vesicular cells, localized rupture of the outer membranes, and inhibition of xylem growth (Figure 2A). Scanning electron microscopy showed that, due to BS2-6 infestation, mycelia and conidia were seen in individual leaf cells. This infection impeded water and nutrient transport and blocked the leaf stomata, which led to the disintegration of leaf tissues (Figure 2E–G). In contrast, the healthy leaves exhibited a normal tissue structure, intact cell walls, and visible shapes of the chloroplasts in their leaf cross-sections (Figure 2B–D).

### 3.3. Physiological Response of F. oxysporum BS2-6 In Vitro

**Figure 3:**
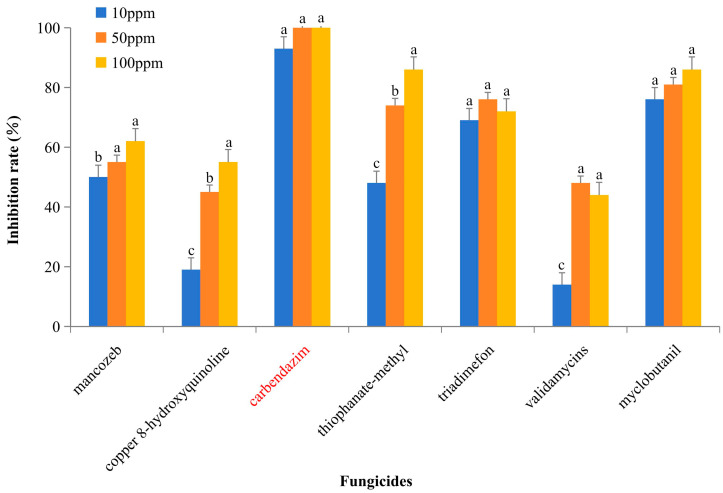
Inhibition rate (%) of chemical compounds on BS2-6 in vitro. Data are presented as the mean of three independent experiments ± SD. Note: the red txt represents carbendazim; the letters abc represent significant differences in the data, with the same letters indicating no significant difference and different letters indicating a significant difference.

We tested seven compounds at three concentrations (100 ppm, 50 ppm, and 10 ppm) for their ability to inhibit the mycelial growth of BS2-6. Among these tested compounds, carbendazim proved to be the most effective. It completely inhibits the mycelial growth at both 100 ppm and 50 ppm and reduces growth by 93.15% at 10 ppm. Four fungicides, mancozeb, thiophanate-methyl, triadimefon, and myclobutanil, inhibited mycelial growth by more than 50% at all tested concentrations. Among them, satistically significant differences were observed between mancozeb and thiophanate-methyl. In contrast, two antibacterial compounds, copper 8-hydroxyquinoline and validamycins, showed no effect on the mycelial growth at 10 ppm and only partially inhibited growth at the concentrations of 50 ppm and 100 ppm (Figure 3).

### 3.4. Sequencing, Assembly, and Evaluation

We performed long reads of the 20 kb library, which generated 4.49 Gb of subreads with a sequencing depth of 86.8×. Furthermore, short reads produced a total of 11.37 Gb subreads, with a sequencing depth of 219.8×. However, transcriptome sequencing yielded 17.45 Gb of subreads, which were utilized for gene annotation and expression analysis (Table S1). The genome assembly resulted in 31 contigs, with a contig N50 length of 2.80 Mb and a genome size of 51.73 Mb, with 47.37% GC content. The BUSCO evaluation showed that BS2-6 assembled 289 complete direct homologous single-copy genes (99.66%) (Table 1). Across the RNA-seq reads, 94.75% to 98.11% were mapped successfully to the genome (Table S2). These results suggest that the *F. oxysporum* BS2-6 genome assembly is highly accurate and complete.

### 3.5. Genome Annotation

A repetitive sequence prediction of BS2-6 identified 2.95 Mb of repetitive sequences (5.70% of the whole genome proportion), containing 17 different classes. Among them, the ClassII/TIR class accounted for the longest repetitive sequence (1.09 Mb) and the unknown class accounted for the highest number of genes (1820) (Table S3). Gene prediction showed that the prediction was based on the *ab* initio strategy, which predicted up to 14,877 genes, while the homology strategy predicted up to 14,907 genes, and the Unigene strategy predicted up to 17,684 genes. The integration of the results above led to a final prediction of 15,794 genes, with an average gene length of 1.6 kb and a gene density of 305 genes per Mb (Table 2).

The prediction of non-coding RNAs showed the presence of 338 tRNAs (2.14%), 96 rRNAs (0.61%), and 42 other ncRNAs (0.27%). Additionally, the BLAT comparison identified 27 pseudogenes (0.17%). A comparison of the annotations with functional databases showed that BS2-6 was annotated with 8898 genes (56.34%) in the GO database, 7339 genes (46.47%) in the KOG database, 3774 genes (23.90%) in the KEGG database, and 11,329 genes (71.73%) in the Pfam database. Furthermore, 8929 genes (56.53%) were annotated in Swiss-Prot database, 15,729 genes (99.59%) were annotated in the TrEMBL database, and 15,729 genes (99.59%) were annotated in the Nr database. Dedicated database annotations indicated that BS2-6 contained 917 genes (5.81%) in the CAZyme database, 5088 genes (32.21%) in the PHI database, 154 genes (0.98%) in the TCDB database, along with 1243 secreted proteins (7.87%) and 250 effector proteins (1.58%) (Table 3). Additionally, 264 transcription factors (TFs) were identified in the BS2-6 genome (Table S4).

### 3.6. Phylogenetic Evolution

**Figure 4:**
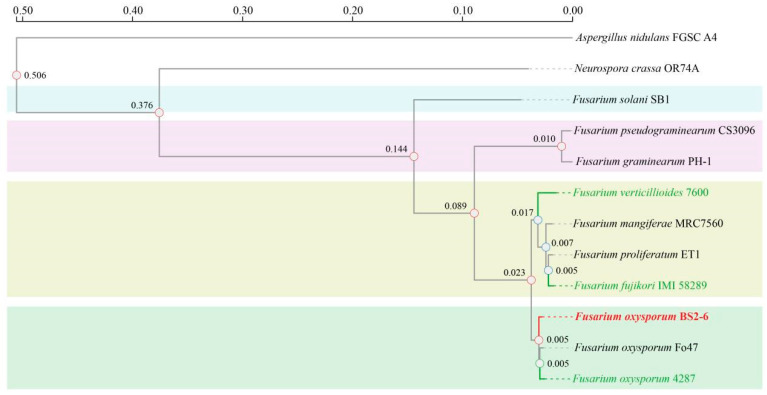
Maximum likelihood tree showing phylogenetic relationships of *Fusarium* species.

The genome-wide phylogenetic tree shows that *F. oxysporum* BS2-6 is most closely related to *F. oxysporum* Fo47 and *F. oxysporum* FOL4287, all of which belong to the same *F. oxysporum* species. The phylogenetic relationships among other *Fusarium* species were in agreement with those previously reported [9,57] (Figure 4). It is noteworthy that FOL4287 has a high repeat sequence of 16.83 Mb, accounting for 27.41% of the whole genome [9], while BS2-6 only has a repeat sequence of 2.95 Mb, accounting for 5.70% of the genome-wide proportion (Table S3). This discrepancy may be attributed to varying rates of gene loss during the genome-wide evolution of *Fusarium* species.

### 3.7. Comparative Genome Analysis

**Figure 5:**
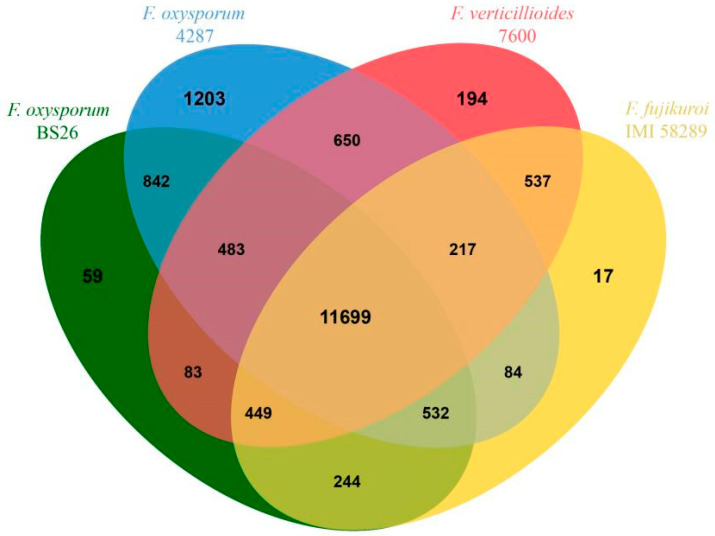
Venn diagram of orthologous gene families among BS2-6 and other three species (Ff.IMI58289, Fv.7600, and FOL4287).

We used the genome of tomato wilt strain FOL4287, the first sequenced and characterized genome of *F. oxysporum*, for a comparative analysis with the BS2-6 genomes. When mapped to the reference genome, only 87.35% of the reads were mapped on nine chromosomes of FOL4287 (Chr #4, #5, #7, #8, #9, #10, #11, #12, and #13). The other six Fol lineage-specific (Fol LS) chromosome regions of FOL4287 (Chr #3, #6, #14, #15, and parts of chr #1 and #2) [9] were poorly mapped. Furthermore, five contigs (contig17, contig18, contig19, contig20, and contig24) contained BS2-6-specific sequences (Figure S1). In total, 11,699 gene families were shared among the four *Fusarium* species. Of these, 59 gene families were found only in the BS2-6 genome, while FOL4287 contained 1203 unique families, Fv.7600 had 194 unique families, and Ff.IMI58289 had 17 unique families (Figure 5).

### 3.8. Identification of Secondary Metabolite Gene Families

**Figure 6:**
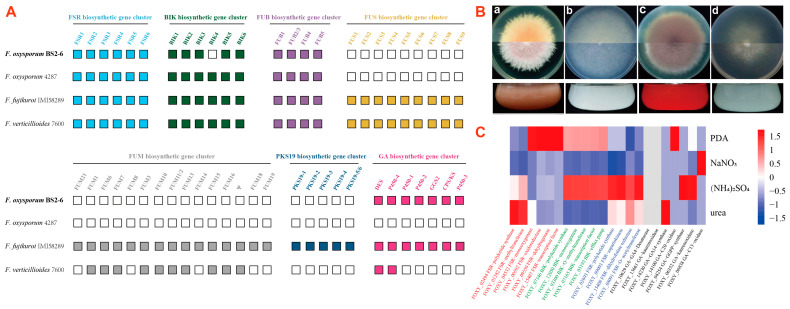
(**A**) Comparison of FSR, BIK, FUB, FUS, FUM, PKS19, and GA biosynthetic gene clusters between four *Fusarium* species (Fo.BS2-6, Ff.IMI58289, Fv.7600, and FOL4287). (**B**) Phenotypic and pigmentation characteristics of *F. oxysporum* BS2-6. Colony morphology of the strains grown on PDA medium (**a**), solidified Czapek medium containing NaNO_3_ (**b**), (NH_4_)_2_SO_4_ (**c**), urea, (**d**) and the variation in the pigmentation of the strains. (**C**) Heatmap of expression data derived from RNA-Seq for the core enzyme gene cluster after culturing in different nitrogen availability amounts. Note: FSR—Fusarubin, BIK—bikaverin, FUB—Fusaric acid, FUS—fusarin C, FUM—fumonisin, PKS19—fujikurin, GA—gibberellin, and Ψ indicates the pseudogene. The white box represents no detected cluster genes.

Secondary metabolite (SM) biosynthetic gene families comprise mainly polyketide synthase (PKS), non-ribosomal peptide synthetase (NRPS), dimethylallyl tryptophan synthase (DMATS), terpene cyclase (TC), cytokinin biosynthetic genes, and auxin biosynthetic genes. This analysis is based on BS2-6 along with three reference genomes (Ff.IMI58289, FV.7600, and FOL4287). The results show that complete FSR and FUB biosynthetic gene clusters are detected in all four *Fusarium* genomes, while *BIK4* of the BIK cluster is absent from BS2-6. Meanwhile, BS2-6 lacks the complete FUS, FUM, and PKS19 biosynthetic gene clusters, indicating its inability to synthesize SMs, such as Fusarin C, Fumonisin, and Fujikurins. Similarly, FOL4287 also lacks these complete gene clusters and a complete GA biosynthetic gene cluster is missing. This GA biosynthetic gene cluster contains seven functional genes (*DES*, *P450-4*, *P450-1*, *P450-2*, *GGS2*, *CPS/KS*, and *P450-3*) that are essential for gibberellin production (Figure 6A, Table S5).

### 3.9. Broad Spectrum Secondary Metabolite Production

We found that BS2-6 showed differences in colony morphology and pigmentation, when grown on solid and liquid media. This indicates that they have metabolic variations between them (Figure 6B). To verify the expression of the SM biosynthetic genes in BS2-6, we cultivated the strains under four standardized culture conditions (PDA, NaNO_3_, (NH_4_)_2_SO_4_, and urea). The optimal conditions for the production of the different metabolites were previously shown to vary considerably regarding nitrogen availability in BS2-6 [11]. We compared the metabolite production levels with the transcriptome profiles of SM biosynthetic genes generated by RNA-seq for these four conditions.

The results indicate that the expression of the Fusarubin gene in strain BS2-6 was low under all four growth conditions, consistent with the results already reported for Fusarubin production under alkaline conditions [57]. In contrast, the strain exhibited a high expression of the BIK gene in all PDA conditions, with especially high levels under (NH_4_)_2_SO_4_ conditions. For Fusaric acid, the strains maintained a high expression level under all four conditions, with the highest expression under (NH_4_)_2_SO_4_. Previously, the regulation of GA biosynthesis has been extensively studied for strain Ff.IMI58289. It has been shown that GA gene expression is strictly regulated by nitrogen availability. However, other *Fusarium* species were not reported to have ability to produce GA. To determine if different conditions are optimal for GA production in BS2-6, we analyzed the expression by RNA seq data. Notably, strain BS2-6 exhibited no expression of GA genes, despite possessing a complete GA gene cluster in its genome (Figure 6C). In addition, the expression of other SMs genes is mentioned in Table S6.

### 3.10. Virulence Assay on Sugarcane

We conducted leaf and stem infection tests on 1-month-old and 5-month-old sugarcane to evaluate the virulence differences under different nitrogen sources. This approach allowed us to evaluate how different nitrogen sources influence the infection of sugarcane with PBD. Our results indicate that the incidence of PBD is the highest under PDA and (NH_4_)_2_SO_4_ conditions, while it is the lowest under the urea condition. Furthermore, expression levels of secondary metabolites were also evaluated to determine their association with disease severity [2]. Additionally, the expression levels of SMs under these different conditions suggest a close relationship between the incidence and severity of PBD and toxins produced by BS2-6 (Table S7).

## 4. Discussion

*Fusarium oxysporum*, is a pathogenic fungus commonly found in soils that causes a deadly vascular wilting and rotting syndrome in plants around the world [58,59]. The symptoms include the chlorosis of leaves, necrosis of the vascular system, general wilting, rotting, and the death of the colonized plant. More than 120 known strains or ‘special forms’ (formae speciales; f. sp.) of *F. oxysporum* are specific to a unique host plant [60,61]. For example, *F. oxysporum* f. sp. *cubense* in particular poses an urgent threat to bananas, causing the deadly Panama disease [62]. These formae speciales characterized by host specificity render the *F. oxysporum* species complex an ideal model for studying the intricate relationships between pathogenicity, virulence, and host specificity. In our previous study, we were the first to report that *F. oxysporum* causes sugarcane PBD in China [3]. Based on the observed disease incidence and severity, *F. oxysporum* induced the top rot of sugarcane more rapidly than the previously reported pathogens, *F. veticillioides* and *F. proliferatum*, and led to the death of the plant in a shorter time [1,2].

In this study, the cellular morphology of *F. oxysporum*-infected leaf tissues was observed for the first time using microscopic techniques, and it was found that *F. oxysporum* did not cause the death of epidermal cells, but significant necrosis occurred in the chloroplasts. *F. oxysporum* enters from the stomata into the leaves, prompting the production of more water between the leaf cells that creates a favorable environment for its reproduction and accelerating the infestation of leaves. Additionally, *F. oxysporum* secretes various types of toxins and hormones, which promotes the disintegration of the cell wall, separation of the plasma membrane, and rupture of the cell membrane, just like the response of rice leaves to rice blast invasion [63].

We obtained a high-quality genome sequence of *F. oxysporum* BS2-6 by combining PacBio long-read sequencing with the Illumina short-read error correction and mapped to the reference genome of *F. oxysporum* f. sp. *lycopersici* 4287 (FOL4287). The BS2-6 genome of 51.73 Mb with a GC content of 47.37% was assembled and well mapped on nine core chromosomes (Chr #4, #5, #7, #8, #9, #10, #11, #12, and #13), except six Fol lineage-specific (Fol LS) chromosome regions of FOL4287 (Chr #3, #6, #14, #15, and parts of chr #1 and #2). Based on this assembly, we conducted a gene prediction and functional annotation. We found that the BS2-6 genome contains 2.95 Mb of repetitive sequences, accounting for 5.70% of the genome-wide proportion. These repetitive sequences in the genome provide raw materials for species evolution and indirectly promote the differentiation of species. Additionally, multi-copy repetitive sequences are closely related to chromosome level transfer and rapid differentiation [9]. Moreover, we identified the large number of secondary metabolite biosynthetic gene families from the sequenced genomes. These gene families mediate the production of toxins or hormones that directly inhibit photosynthesis, causing changes in the water metabolism of the host sugarcane, resulting in wilting and even rotting. They also indirectly affect phenolic metabolism, causing the necrosis of the sugarcane leaves [57].

**Figure 7:**
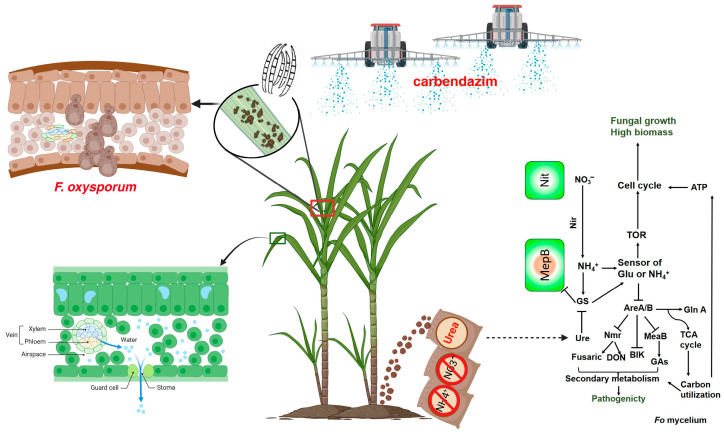
The schematic figure illustrates the intricate relations between the sugarcane and pathogen *F. oxysporum* BS2-6. Schematic diagram of nitrogen fertilizer application and chemical prevention and control technology.

It was discovered that sugarcane PBD develops under conditions of excessive nitrogen fertilization and optimal growth environments [56]. We performed RNA-seq to analyze the response of BS2-6 to various chemical compounds. We cultured BS2-6 in the medium with ammonium sulfate, sodium nitrate, and urea as nitrogen sources. It was found that ammonium sulfate promoted the mycelial growth and pigment secretion of BS2-6, and increased the expression of some genes related to the synthesis of secondary metabolites. Based on the RNA seq data, we hypothesized that the up-regulated expression of the ammonium transporter member (*AMT1*) enhances the uptake of NH_4_^+^ and its transmembrane transport. Additionally, the involvement of *MepB* in NH_4_^+^ uptake and signaling, along with the increased expression of *GS* and GDH, promotes the mycelial growth and sporulation of BS2-6, leading to the aggravation of sugarcane pathogenesis [11,64] (Figure 7).

Field experiments were conducted to analyze the effects of different N fertilizer applications on the occurrence and quality of sugarcane PBD. Our data indicate that excessive nitrogen application increases both the incidence and severity of sugarcane PBD. Furthermore, the incidence of PBD was significantly higher with the application of ammoniacal nitrogen (ammonium sulphate) compared to the other forms of nitrogen, such as nitrate and urea. This demonstrates that the excessive and inappropriate use of nitrogen fertilizer exacerbates the incidence of PBD [11,64]. It is recommended to use urea as the nitrogen fertilizer for sugarcane growth, as it can effectively provide the necessary nitrogen amount and reduce the occurrence of sugarcane PBD. Additionally, our study found that among various commonly used fungicides and chemicals, carbendazim applied at concentrations of 10 ppm, 50 ppm, or 100 ppm can interfere with the cell division of *F. oxysporum*, thereby inhibiting the growth and reproduction of the pathogen. This can help reduce or prevent the occurrence and spread of PBD. It has been demonstrated that *Trichoderma* sp., as a widely used microbial agent, exhibits remarkable effects in disease prevention, insect control, and growth promotion, making it highly used in sugarcane production. Specifically, it has been proven to manage diseases effectively, thereby reducing the incidence of diseases and enhancing sugarcane’s resistance to drought and cold.

Our results highlight effective strategies for preventing and controlling PBD during sugarcane production. First, select urea as the nitrogen fertilizer for application, while avoiding excessive application to improve the sugarcane’s resistance to the disease. Before sowing, soak the cane stems in a 0.2% carbendazim solution for 30 min to effectively eliminate the pathogenic fungi on their surface and enhance germination rates. Early on in the PBD outbreak, apply chemical agents, such as carbendazim every 10–15 days, continuing for 2 to 3 applications. Additionally, dilute *Trichoderma* sp. in water and stir until completely mixed. Then, apply it uniformly to the leaves, stems, and root systems of the sugarcane plants using a sprayer. The recommended application rate is 200 to 500 g per acre. Implementing these measures can significantly reduce the occurrence and spread of sugarcane PBD, ensuring healthy sugarcane growth. Additionally, whole-genome sequencing data will aid in exploring the disease pathogenicity and eventually developing resistant sugarcane cultivars.

## 5. Conclusions

In this study, we selected the representative strain BS2-6 to perform morphological observations of colonies and assess pathogenicity. We also examined the effects of BS2-6 infestation on the ultrastructure of sugarcane leaves. Additionally, we sequenced the whole genome of BS2-6 and analyzed its growth and pathogenicity under varying nitrogen sources, as well as the inhibitory effects of different chemical reagents on its growth. These findings provide valuable insights for the effective prevention and control of PBD during sugarcane production. Our research establishes a foundation for developing a sustainable system for disease prevention and control technology system for sugarcane disease. In the future, we will continue to explore the relationship between saprophytism and parasitism from a whole genome perspective, which will provide insights into the mechanisms regulating life-style transitions in the *Fusarium* species complex that causes sugarcane PBD.

## Figures and Tables

**Table 1:** Global statistics for the *F. oxysporum* BS2-6 genome assembly and BUSCO analysis.

Summary	BS2-6
Genome size (bp)	51,731,010
Contig number	31
Contig N50 (bp)	2,803,775
Contig N90 (bp)	1,130,085
Contig max (bp)	5,230,794
GC content	47.37%
Accession No.	JALBXV000000000
Complete BUSCOs (C)	289 (99.66%)
Complete and single-copy BUSCOs (S)	288 (99.31%)
Complete and duplicated BUSCOs (D)	1 (0.34%)
Fragmented BUSCOs (F)	1 (0.34%)
Missing BUSCOs (M)	0
Total BUSCO groups searched	290

**Table 2:** Functional annotation of predicted protein-coding genes in *F. oxysporum* BS2-6.

Method	Software		BS2-6
*Ab* initio	Augustus	-	12,255
	Glimmer HMM	-	14,455
	SNAP	-	14,877
Homolog-based	GeMoMa	*F*. *fujikuroi* IMI58289	14,581
		*F. fujikuroi* KSU3368	14,907
		*F*. *fujikuroi* FGSC8932	14,544
Unigene	PASA	-	17,684
Integration	EVM	-	15,794
Gene density (number of genes per Mb)			305
Average gene length (Kb)			1.6
Total exon length (Mb)			22.91
Total intron length (Mb)			2.5
Exons			45,417
Average exon length (bp)			504.4
Introns			29,623
Average intron length (bp)			84.29

**Table 3:** Statistics of the function database and dedicated database annotation results of BS2-6.

Database	*F. oxysporum* BS2-6
GO	8898 (56.34%)
KOG	7339 (46.47%)
KEGG	3774 (23.90%)
Pfam	11,329 (71.73%)
Swiss-Prot	8929 (56.53%)
TrEMBL	15,729 (99.59%)
Nr	15,729 (99.59%)
CAZyme	917 (5.81%)
PHI	5088 (32.21%)
TCDB	154 (0.98%)
SP	1243 (7.87%)
EP	250 (1.58%)
tRNA	338 (2.14%)
rRNA	96 (0.61%)
Other ncRNA	42 (0.27%)
Pseudogene	27 (0.17%)

Note: CAZyme: carbohydrate-active enzymes database, PHI: pathogen–host interaction, TCDB: transporter classification database, SP: secreted protein, EP: effector protein.

## Data Availability

All data generated or analyzed during this study were included in this published article and its Supplementary Information files. Further inquiries can be directed to the corresponding authors.

## Supplementary Materials

The following supporting information can be downloaded at: https://www.mdpi.com/article/10.3390/microorganisms13030573/s1, Figure S1: Genomic alignment between *F. oxysporum* BS2-6 and FOL4287; Table S1: Summary of sequencing data generated in this study; Table S2: Summary the RNA-Seq data of *F. oxysporum* BS2-6; Table S3: Repeat content result of the genome sequence of *F. oxysporum*; Table S4: The statistical results of transcription factors identified in *F. oxysporum* BS2-6; Table S5: Presence of secondary metabolite gene clusters in the analyzed *Fusarium* strains; Table S6: Expression of the secondary metabolite key enzyme encoding genes under PDA and difference nitrogen conditions; Table S7: The pathogenicity of strain BS2-6 to sugarcane.
