# Supplementary material for: Characterization and Genome Analysis of Fusarium oxysporum Provides Insights into the Pathogenic Mechanisms of the Pokkah Boeng Disease in China
Source: Microorganisms. 2025 Mar 3;13(3):573. doi: 10.3390/microorganisms13030573 (PMC11944933; doi:10.3390/microorganisms13030573)
Supplement: Supplementary file 1 [file microorganisms-13-00573-s001.zip › microorganisms-3499549-supplementary.pdf]

Supplementary Figures:

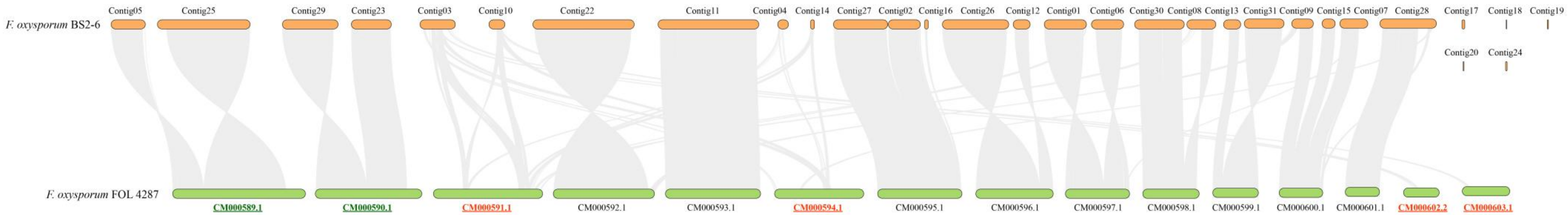

Figure S1. Genomic alignment between *F. oxysporum* BS2-6 and FOL4287.

Note: Synteny between homologous chromosomes were shown by gray areas.

Supplementary Tables:

Table S1. Summary of sequencing data generated in this study.

Types	Platform	Clean base	Depth	Application
Long reads	PacBio Sequel	4.49 Gb	86.8 ×	Genome assembly
Short reads	Illumina Hi-seq	11.37 Gb	219.8 ×	Genome survey and base level correction
RNA-Seq	Illumina Nova-seq	17.45 Gb	-	Gene annotation and expression analysis

Table S2. Summary the RNA-Seq data of *F. oxysporum* BS2-6.

ID	Medium	Total reads	Mapped reads	GC(%)	≥Q30(%)	Accession No.
FO-PDA	PDA	32,429,514	31,817,186 (98.11%)	52.41	92.49	SRR18233033
FO-NA	Czapek (sodium nitrate)	27,747,406	27,207,878 (98.06%)	52.20	92.21	SRR18233041
FO-UR	Czapek (urea)	24,987,292	23,676,119 (94.75%)	51.89	92.53	SRR18233040
FO-NH4	Czapek (ammonium sulfate)	31,874,058	31,078,053 (97.50%)	52.40	92.62	SRR18233036

Table S3. Repeat content result of the genome sequence of *F. oxysporum*.

Type	*F. oxysporum* BS2-6		
	**Number**	**Length (bp)**	**Percent (%)**
ClassI/DIRS	31	33,443	0.06
ClassI/LINE	200	221,467	0.43
ClassI/LTR	713	749,430	1.45
ClassI/LTR/Copia	42	14,438	0.03
ClassI/LTR/Gypsy	54	9,992	0.02
ClassI/PLE丨LARD	50	92,482	0.18
ClassI/SINE	150	29,904	0.06
ClassI/TRIM	1	2,165	0.00
ClassI/Unknown	29	13,884	0.03
ClassII/Crypton	1	57	0.00
ClassII/Helitron	2	73	0.00
ClassII/MITE	644	120,151	0.23
ClassII/TIR	1,293	1,093,933	2.11
ClassII/Unknown	55	40,130	0.08
PotentialHostGene	28	24,533	0.05
SSR	607	219,870	0.43
Unknown	1,820	371,001	0.72
**Total**	**3,900**	**2,950,420**	**5.70**

Table S4. The statistical results of transcription factors identified in *F. oxysporum* BS2-6.

**Families**	**Number**	**Families**	**Number**
AP-2	3	NF-YB	3
ARID	3	NF-YC	3
bHLH	21	Others	5
COE	2	PC4	1
CP2	1	RFX	1
DACH	2	SRF	2
Fork head	4	TEA	1
HMG	9	TSC22	2
HMGA	3	ZBTB	37
Homeobox	12	zf-BED	2
HSF	3	zf-C2H2	83
HTH	3	zf-CCCH	13
MYB	20	zf-GATA	13
NDT80_PhoG	3	zf-LITAF-like	5
NF-YA	1	zf-MIZ	3
**Total**			**264**

Table S5. Presence of secondary metabolite gene clusters in the analyzed Fusarium strains.

Secondary metabolitea	Key enzyme	*F. oxysporum* BS2-6	*F. fujikuroi* IMI 58289	*F. verticillioides* 7600	*F. oxysporum* 4287	Annotated
**Polyketide synthases**						
Equisetin	PKS 1	FOXY_00305	FFUJ_02219	FVEG_12610	FOXG_15296	probable polyketide synthase
	PKS 2	n.p.	FFUJ_00118	FVEG_00079	n.p.	polyketide synthase
Fusarubin	PKS 3	FOXY_02494	FFUJ_03984	FVEG_03696	FOXG_05816	fusarubin cluster-polyketide synthase
		FOXY_01282	FFUJ_03985	FVEG_03697	FOXG_05817	fusarubin cluster-methyltransferase
		FOXY_04532	FFUJ_03986	FVEG_03698	FOXG_05818	fusarubin cluster-monooxygenase
		FOXY_00592	FFUJ_03987	FVEG_03699	FOXG_05819	fusarubin cluster-oxidoreductase
		FOXY_09550	FFUJ_03988	FVEG_03700	FOXG_05820	fusarubin cluster-dehydrogenase
		FOXY_15407	FFUJ_03989	FVEG_03701	FOXG_05821	fusarubin cluster-transcription factor
Bikaverin	PKS 4	FOXY_07146	FFUJ_06742	FVEG_03379	FOXG_04757	bikaverin cluster-polyketide synthase
		FOXY_12050	FFUJ_06743	FVEG_03380	FOXG_04756	bikaverin cluster-monooxygenase
		FOXY_03300	FFUJ_06744	FVEG_03381	FOXG_04755	bikaverin cluster-O-methyltransferase
		n.p.	FFUJ_06745	FVEG_03383	FOXG_04754	bikaverin cluster-transcription factor
		FOXY_07165	FFUJ_06746	FVEG_03382	FOXG_04753	bikaverin cluster-transcription factor
		FOXY_05193	FFUJ_06747	FVEG_03384	FOXG_04752	bikaverin cluster-efflux pump
	PKS 5	FOXY_15308	n.p.	n.p.	FOXG_10805	probable polyketide synthase
Fusaric acid	PKS 6	FOXY_02693	FFUJ_02105	FVEG_12523	FOXG_15248	polyketide synthase
		FOXY_09093	FFUJ_02106<br>FFUJ_02107	FVEG_12522<br>FVEG_12521	FOXG_15247	uncharacterized protein
		FOXY_15408	FFUJ_02108	FVEG_12520	FOXG_15244	Dihydrofolate reductase
		FOXY_08091	FFUJ_02109	FVEG_12519	FOXG_15243	homoserine O-acetyltransferase
Fujikurins	PKS 7	FOXY_15316	FFUJ_06260	FVEG_01914	FOXG_03051	polyketide synthase
	PKS 8	n.p.	FFUJ_12090	FVEG_10497	FOXG_11892	polyketide synthase-nonribosomal peptide
	PKS 9	FOXY_06461	FFUJ_14695	FVEG_11932	FOXG_03945	related to polyketide synthase
Fusarin C	PKS 10	n.p.	FFUJ_10058	FVEG_11086	n.p.	fusarin C cluster-polyketide synthase
			FFUJ_10057	FVEG_11085		fusarin C cluster-hydrolase
			FFUJ_10056	FVEG_11084		fusarin C cluster-elongation factor 1
			FFUJ_10055	FVEG_11083		fusarin C cluster-peptidase
			FFUJ_10054	FVEG_11082		fusarin C cluster-oxidoreductase

			FFUJ_10053	FVEG_11081		fusarin C cluster-probable transporter
			FFUJ_10052	FVEG_11080		fusarin C cluster-oxidoreductase
			FFUJ_10051	FVEG_11079		fusarin C cluster-cytochrome P450
			FFUJ_10050	FVEG_11078		fusarin C cluster-methyltransferase
			FFUJ_09240	n.p.		fumonisin biosynthetic transcription factor
			FFUJ_09241	FVEG_00316		fumonisin cluster-peptidsynthase
			FFUJ_09242	FVEG_00317		fumonisin cluster-P450 monooxygenase
			FFUJ_09243	FVEG_00319		fumonisin cluster-dehydrogenase
			FFUJ_09244	n.p.		fumonisin cluster-aminotransferase
			FFUJ_09245	FVEG_00320		fumonisin cluster-dioxygenase
			FFUJ_09246	FVEG_00321		fumonisin cluster-fatty acyl-CoA Synthase
Fumonisin	PKS 11	n.p.	FFUJ_09247	FVEG_00323	n.p.	fumonisin cluster-P450 monooxygenase
			FFUJ_09248	FVEG_00324		
			FFUJ_09249	FVEG_00325		fumonisin cluster-Dehydrogenase
			FFUJ_09250	n.p.		fumonisin cluster-peptidsynthase
			FFUJ_09251	FVEG_00326		fumonisin cluster-P450 monooxygenase
			FFUJ_09252	FVEG_00328		fumonisin cluster-fatty acyl-CoA Synthase
			FFUJ_09253	FVEG_00329		fumonisin cluster-longevity assurance
			FFUJ_09254	n.p.		fumonisin biosynthetic ABC transporter
			FFUJ_09255	n.p.		Uncharacterized protein
	PKS 12	FOXY_09893	FFUJ_10347	FVEG_13715	FOXG_16513	probable polyketide synthase
	PKS 12a-1	n.p.	n.p.	n.p.	FOXG_14850	probable polyketide synthase
	PKS 12a-2	n.p.	n.p.	n.p.	FOXG_15886	probable polyketide synthase
Gibepyrones	PKS 13	FOXY_09017	FFUJ_12020	FVEG_10535	FOXG_11954	polyketide synthase
	PKS 14	n.p.	FFUJ_11034	FVEG_08425	FOXG_10070	probable polyketide synthase
	PKS 15	n.p.	n.p.	FVEG_05537	n.p.	probable polyketide synthase
	PKS 16	n.p.	FFUJ_11199	n.p.	n.p.	probable type I polyketide synthase
	PKS 17	n.p.	FFUJ_12066	n.p.	n.p.	probable type I polyketide synthase
Asperfuranone	PKS 18	n.p.	FFUJ_12074	n.p.	n.p.	polyketide synthase
Fujikurins	PKS 19	n.p.	FFUJ_12239	n.p.	n.p.	probable polyketide synthase

			FFUJ_12240			related to C.carbonum toxD protein
			FFUJ_12241			related to DltD N-terminal domain protein
			FFUJ_12242			related to monocarboxylate transporter 2
			FFUJ_12243			O-methylsterigmatocystin
			FFUJ_12244			oxidoreductase
	PKS 20	FOXY_00579	FFUJ_12707	FVEG_13420	FOXG_14587	probable polyketide synthase
Depudecin	PKS 39	FOXY_12411	n.p.	FVEG_01736	FOXG_02884	probable polyketide synthase
	PKS 41	n.p.	n.p.	n.p.	FOXG_02741	probable polyketide synthase
**Non-ribosomal peptide synthetases**						
Malonichrom	NRPS 1	FOXY_14210	n.p.	FVEG_12503	FOXG_17422	Nonribosomal peptide synthetase
Ferricrocin	NRPS 2	FOXY_10886	FFUJ_04614	FVEG_04296	FOXG_06448	non-ribosomal peptide synthetase
	NRPS 3	FOXY_12120	FFUJ_06929	FVEG_03243	FOXG_04898	non-ribosomal peptide synthetase
	NRPS 4	FOXY_11382	FFUJ_08113	FVEG_11762	FOXG_13024	related to non-ribosomal peptide synthetase
Fusarinine	NRPS 6	FOXY_10399	FFUJ_10736	FVEG_08697	FOXG_09785	related to AM-toxin synthetase (AMT)
	NRPS 10	FOXY_04277	FFUJ_03506	FVEG_05643	FOXG_02458	alpha-aminoadipate reductase large subunit
	NRPS 11	FOXY_11726	FFUJ_10934	FVEG_08516	FOXG_09998	non-ribosomal peptide synthetase
	NRPS 12	FOXY_08920	FFUJ_14790	FVEG_11841	FOXG_13405	non-ribosomal peptide synthetase
	NRPS 13	FOXY_15321	FFUJ_02440	FVEG_07777	FOXG_01411	non-ribosomal peptide synthetase
	NRPS 17	n.p.	FFUJ_03641	FVEG_14029	n.p.	AM-toxin synthetase (AMT)
Fusaridione A	NRPS 20	FOXY_13720	FFUJ_06720	FVEG_03415	FOXG_04709	related to AM-toxin synthetase (AMT)
	NRPS 21	FOXY_03620	FFUJ_02022	FVEG_09864	FOXG_10932	non-ribosomal peptide synthetase
Beauvericin	NRPS 22	FOXY_07887	FFUJ_09296	n.p.	FOXG_11847	related to non-ribosomal peptide synthetase
	NRPS 23	FOXY_08217	FFUJ_12008	FVEG_10547	FOXG_11967	related to non-ribosomal peptide synthetase
	NRPS 24	n.p.	n.p.	FVEG_06502	n.p.	non-ribosomal peptide synthetase
Acetylaranotin	NRPS 25	n.p.	FFUJ_05347	FVEG_13313	n.p.	related to non-ribosomal peptide synthetase
	NRPS 26	n.p.	n.p.	FVEG_06496	n.p.	non-ribosomal peptide synthetase

Apicidin F	NRPS 31	n.p.	FFUJ_00003	n.p.	n.p.	non-ribosomal peptide synthetase
Ferrirhodin-type	NRPS 32	n.p.	n.p.	n.p.	FOXG_17272	non-ribosomal peptide synthetase
	AAR1	FOXY_06523	FFUJ_02913	FVEG_08245	FOXG_01867	alpha-aminoadipate reductase
**Dimethylallytryptophane synthases**						
r-N-DMAT	DMATS1	FOXY_01547	FFUJ_09179	FVEG_09966	n.p.	tryptophan dimethylallyltransferase
	DMATS3	FOXY_08941	FFUJ_14683	n.p.	FOXG_03930	Uncharacterized protein
	DMATS4	FOXY_15172	n.p.	FVEG_12218	FOXG_13322	tryptophan dimethylallyltransferase
**Terpene cyclases**						
Phytoene	TeTC1	FOXY_14282	FFUJ_11802	FVEG_10718	FOXG_12144	probable geranylgeranyl-diphosphate geranylgeranyltransferase (AL-2)
		FOXY_10629	FFUJ_14331	FVEG_09398		gibberellin cluster-GA4-Desaturase
		FOXY_13661	FFUJ_14332	FVEG_16575		gibberellin cluster-kaurenoxidase
		FOXY_14230	FFUJ_14333			gibberellin cluster-GA14-synthase
Gibberellins	DTC1	FOXY_14100	FFUJ_14334		n.p.	gibberellin cluster-C20-oxidase
		FOXY_04254	FFUJ_14335	n.p.		gibberellin cluster-GGPP-synthase
		FOXY_06352	FFUJ_14336			gibberellin cluster-kaurenoxidase
		FOXY_06838	FFUJ_14337			gibberellin cluster-C13-oxidase
	STC1	FOXY_02367	FFUJ_00036	FVEG_00016	n.p.	Presilphiperfolan-8-beta-ol synthase
	STC2	FOXY_01334	FFUJ_00969	FVEG_00910	n.p.	related to terpene synthase
(+)-Eremophilene	STC3	FOXY_05965	FFUJ_04067	FVEG_03771	FOXG_05900	related to pentalenene synthase
(+)-Koraiol	STC4	FOXY_13155	FFUJ_10353	FVEG_13707	FOXG_16522	uncharacterized protein
(–)-Guaia-6,10(14)	STC5	FOXY_13875	FFUJ_11739	n.p.	FOXG_12225	Presilphiperfolan-8-beta-ol synthase
(–)-α-Acorenol	STC6	FOXY_06048	FFUJ_12585	FVEG_13850	FOXG_13565	related to pentalenene synthase
	STC7	FOXY_04773	FFUJ_12026	FVEG_10529	FOXG_11949	related to terpene synthase
	STC8	n.p.	FFUJ_09423	FVEG_10174	n.p.	uncharacterized protein
Longiborneol	STC9	n.p.	n.p.	n.p.	n.p.	related to terpene synthase
**Cytokinin biosynthetic genes**						

Cytokinins	IPT_LOG1	n.p.	FFUJ_03536	FVEG_15697	n.p.	hypothetical protein
	P450-1	FOXY_04309	FFUJ_03535	FVEG_15698	FOXG_01241	pisatin demethylase cytochrome P450
Cytokinins	IPT_LOG2	FOXY_04871	FFUJ_14354	FVEG_09393	FOXG_20358	hypothetical protein
	P450-2	FOXY_10666	FFUJ_14353	FVEG_09392	n.p.	pisatin demethylase cytochrome P450
**Auxin biosynthetic genes**						
Auxins	iaaM	FOXY_10951	FFUJ_03653	FVEG_05765	FOXG_02584	Tryptophan-2-monooxygenase
Auxins	iaaH	FOXY_00885	FFUJ_03654	FVEG_05764	FOXG_02586	Related to amidase family protein

(green shading)	other reference species
n.p.	not present in the genome

Note: a: Secondary metabolite gene predictions are based on InterPro domains, manually validated and corrected based on reports in the literature and on comparative analysis of fusaria. PKS: polyketide synthase, NRPS: non-ribosomal peptide synthetase, AAR: alpha-aminoadipate reductase, DMATS: dimethylallyltryptophan synthase, TC: terpene cyclase.

Table S6. Expression of the secondary metabolite key enzyme encoding genes under PDA and difference nitrogen conditions.

Key enzyme	PDA	$NaNO_3$	urea	$NH_4(SO_4)_2$
PKS 1	3.18	0.46	0.12	0.03
PKS 2	n.p.	n.p.	n.p.	n.p.
PKS 3 Fusarubin	0.18	0.06	1.52	0.64
	0.09	0.00	1.09	0.68
	1.32	0.00	0.46	0.00
	2.07	0.14	0.00	0.22
	5.98	0.11	0.00	0.64
	0.20	0.00	0.00	0.00
PKS 4 Bikaverin	4.87	0.06	0.04	7.08
	6.77	0.18	0.23	9.53
	7.72	0.94	0.47	10.56
	n.p.	n.p.	n.p.	n.p.
	6.36	0.06	0.12	8.54
	9.36	1.22	0.92	11.77
PKS 5	5.80	0.01	0.39	4.03
PKS 6 Fusaric acid	3.63	2.61	4.37	5.81
	3.40	2.23	4.09	6.15
	4.01	4.69	7.13	7.98
	2.82	2.53	4.27	6.20
PKS 7	0.23	2.98	1.68	2.21
PKS 9	2.71	0.17	0.38	1.00
PKS 12	0.00	0.00	0.00	0.00
PKS 13	0.38	0.22	3.58	0.25
PKS 20	0.08	0.15	0.05	0.04
PKS 39	0.00	0.00	0.00	0.00
NRPS 1	0.16	1.51	2.82	0.10
NRPS 2	4.50	2.60	3.27	2.65
NRPS 3	8.11	6.17	6.39	7.49
NRPS 4	2.04	0.14	0.29	0.60
NRPS 6	3.60	4.88	7.20	3.40
NRPS 10	1.60	2.14	3.86	2.94
NRPS 11	0.65	0.11	0.74	0.08
NRPS 12	0.22	0.25	0.08	0.24
NRPS 13	3.49	4.00	4.47	3.39
NRPS 20	0.00	0.00	0.00	0.00
NRPS 21	0.56	0.19	0.58	0.58
NRPS 22	2.18	0.61	0.08	0.14
NRPS 23	3.05	0.03	0.05	0.00
AAR1	0.69	2.43	1.52	3.40
DMATS1	1.19	0.19	0.25	1.38
DMATS3	4.89	3.21	0.17	3.89
DMATS4	3.06	0.46	0.00	0.20
TeTC1	3.81	3.04	1.68	2.36
DTC1 Gibberellins	0.00	0.00	0.00	0.00
	0.00	0.00	0.00	0.00
	0.00	0.00	0.11	0.00
	0.07	0.00	0.00	0.00
	0.00	0.00	0.00	0.61
	0.04	0.00	0.00	0.12
	0.00	0.09	0.00	0.00
STC1	0.45	2.64	1.09	1.41
STC2	0.46	1.17	1.40	2.13
STC3	0.12	0.15	0.93	0.22
STC4	0.90	0.16	0.00	0.09

STC5	0.00	0.00	0.00	0.00
STC6	0.27	0.51	0.40	1.27
STC7	0.00	0.00	0.00	0.00
P450-1	0.00	0.00	0.00	0.00
IPT_LOG2	0.29	0.00	0.00	0.00
P450-2	0.13	0.00	0.00	0.00
iaaM	0.05	0.00	0.77	0.00
iaaH	0.13	0.39	0.32	0.09

Note: n.p.: not present in the genome.

Table S7. The pathogenicity of strain BS2-6 to sugarcane.

**Medium**	**Leaf**		**Stalk**	
	**Incidence rate (%)**	**Condition index**	**Incidence rate (%)**	**Condition index**
PDA	70.00	29.33	96.67	34.67
NaNO3	40.00	12.00	50.00	16.00
$(NH_4)_2SO_4$	53.33	16.00	66.67	21.33
Urea	30.00	9.33	40.00	2.67
